# Effect of physical activity promotion program on adherence to physical exercise among patients with type II diabetes in North Shoa Zone Amhara region: a quasi-experimental study

**DOI:** 10.1186/s12889-023-15642-7

**Published:** 2023-04-19

**Authors:** Akine Eshete, Sadat Mohammed, Sisay Shine, Yosef Eshetie, Yibeltal Assefa, Nigussie Tadesse

**Affiliations:** 1 Department of Public Health, Debre Berhan University, Debre Berhan, Ethiopia; 2 Department of Biomedical Science, Debre Berhan University, Debre Berhan, Ethiopia; 3 School of Public Health, The University of Queensland, Brisbane, Australia; 4 Department of Nursing, Debre Berhan University, Debre Berhan, Ethiopia

**Keywords:** Physical activity Promotion Program, Adherence to recommended physical Exercise, Patients with type II diabetes, North Shoa Zone

## Abstract

**Background:**

Diabetes is a significant global public health issue that necessitates self-management. However, this is difficult to put into practice and requires a new approach. The purpose of this study was to assess the effects of a physical activity promotion program on adherence to recommended physical activity and lessons to improve self-management.

**Methods:**

A quasi-experimental study was conducted from January 2020 to February 2021 at North Shoa Zone Public Hospital. The study enrolled 216 type II diabetic patients from four public hospitals. Data were entered into Epi Data V.3.1 and analyzed using SPSS version 22. Data were presented as means of standard deviations for continuous variables and percentages for categorical variables. Intervention and control groups were compared before and after intervention using independent t-tests. A p-value less than 0.05 was considered significant for all statistical tests.

**Results:**

A total of 216 type II diabetics participated in this study. Physical activity promotion programs increased adherence to the recommended number of days and duration (spending time) of physical activity (p < 0.0001). Participants who engaged in the physical activity promotion program significantly increased the mean scores for exercising moderate-intensity activities and spending time (p < 0.05), walking for at least 10 min continuously and spending time (p < 0.05), exercising moderate-intensity recreational activities and spending time (p < 0.05).There was a significant reduction in mean fasting blood glucose after participating in a physical activity program (p < 0.05).

**Conclusion:**

This study demonstrates that a physical activity promotion program makes a significant difference in patient compliance with recommended physical activity and effectively improves patient glycemic control. Health care providers should integrate physical activity programs into existing systems as a common therapeutic service. Primary care platforms such as health posts and health centers can play a key role in integrating health promotion programs to improve self-management behaviors.

## Background

Diabetes mellitus is a major worldwide public health problem affecting about 9.3% of the global adult population in 2019 and increasing to 12.2 by 2045, especially in middle-income countries with 80.6% [1–4]. Ethiopia has the highest prevalence of diabetes, with a prevalence ranging from 2.0 to 6.5% [5]. This rapid increase in diabetes requires self-management behavior, especially in areas with poor health care coverage. Diabetes self-management includes activities and behaviors that patients undertake to manage and treat their condition [6]. Diabetes requires ongoing care with multifactorial risk reduction strategies. According to the American Diabetes Association (ADA), successful diabetes management requires a systematic approach that supports patient behavior change efforts [7].

Physical activity education is a cost-effective treatment in under-resourced settings [8]. The World Health Organization (WHO) defines physical activity as any muscular contraction of skeletal muscles that results in energy expenditure, including actions performed while playing, working, performing household tasks, engaging in leisure activities and traveling [9, 10].

Nowadays, physical activity has been considered a complementary treatment modality in the management and control of non-communicable diseases (NCDs) [11, 12]. Regular physical activity is the safest and best-tested lifestyle measure to reduce the risk of NCD [13–15]. For example, the incidence of diabetes after exercise intervention was lower in the intervention group (7%) than in the control group (11%)[16]. Many studies have also shown that physical activity lowers fasting blood sugar [17–20]. Furthermore, physical activity education was significantly positively associated with achievement of recommended physical activity guidelines [21, 22]. Several studies found an increased impact of behavioral intervention programs on exercise compliance [23–26].

Despite these benefits, one in four adults (23%) do not meet global recommendations for physical activity for health [9, 14]. Further evidence indicates that 66.7% [27] and 59.9% of diabetic patients do not meet international standards [28]. The American Diabetes Association (ADA) recommends that people with type 2 diabetes (T2DM) do at least 150 min of moderate-to-vigorous aerobic exercise per week. Patients should be encouraged to perform strength training at least 2–3 times per week, reduce time spent in daily sedentary activities, and increase overall daily unstructured physical activity [29, 30].

Physical activity is a low priority lifestyle in Ethiopia [13]. A systematic study found that 50.45% of type II diabetic patients did not follow recommended physical activity [31]. Furthermore, various observational studies have reported nonadherence to recommended physical activity levels ranging from 36 to 64.3% [32–37]. This evidence suggests there are gaps in knowledge and practice regarding recommended levels of physical activity and participation. Efforts to promote physical activity-related diabetes self-management behaviors are therefore recommended as a top priority in Ethiopia.

Physical activity promotion programs are important knowledge enhancement strategies that help individuals meet recommended regular physical activity. Therefore, the aim of this study was to evaluate the effects of a physical activity promotion program on recommended physical activity adherence and glycemic control in type II diabetic patients in North Shoa Zone Amhara Region. Furthermore, this study supports evidence-based decision-making for designing appropriate community-based interventions and provides relevant information for planning and designing strategies to foster change. Additionally, the results of this study will help people with diabetes and their healthcare providers plan appropriate interventions to ensure optimal health.

## Methods

### Study area and period

The study was conducted in North Shoa Zone public hospitals from January 2020 to February 2021. North Shoa is one of the thirteenth zones of the Amhara Regional State located in northern Ethiopia. It has 24 districts and three city administrations. There are thirteenth hospitals and all public hospitals have diabetic follow-up services.

### Study design

A quasi-experimental study was conducted in a randomly selected public hospital. After collecting relevant baseline data from the intervention and control groups, a physical activity promotion program was implemented for six months. In this study, physical activity interventions focused three recommended areas of physical activity including work-related activities, travel to and from places activities, and leisure-time-related activities. The diabetic did at least 150 min of moderate-intensity aerobic exercise per week, and 75 min of vigorous-intensity aerobic exercise per week. Additionally, unstructured daily activities such as housework, dog walking, and gardening are the most common. Patients underwent a 30- to 50-minute exercise education program to ensure adherence to recommended activities.

### Subjects and sample selection

A total of 216 eligible participants were enrolled in the study. Five hundred eight patients were excluded from the study for various reasons. These included 294 patients with other types of diabetes, 97 patients with less than three months of follow-up, and 117 patients with serious complications. The study included consenting patients, aged 20 to 70, with no complications, who stayed for at least six months, and had no intention of leaving. Patients who had other types of diabetes, patients who had disease duration of less than 6 months, refused consent, patient who were unable to participate in interventions based on physician assessment (e.g., acute illness, mental illness, and dementia) and patients with severe visual impairment were excluded from the study.

All samples (216) were divided into intervention (108) and control (108) groups assuming an equal sample distribution. Study participants were randomly assigned to intervention and control groups. Intervention and control groups of diabetic patients were selected from different locations within the zone. Study participants were registered under a specific code but were not informed of their group assignment and thus were unaware of differences between the intervention and control groups. The list of participants and their codes are kept only by the researcher. All groups of study participants were geographically separated to avoid the risk of contamination. Additionally, patients, health care providers, and promoters (health educators) were blinded to the study results to avoid the hawthorn effect.

### Implementation and follow-up of intervention

After collecting relevant baseline data from both groups, a physical activity promotion program was implemented for a period of six months. Two health promoters and one facilitator were recruited for the intervention group and trained on the implementation and packaging of physical activity promotion of program modules. Training focuses primarily on session structure, communication skills and style. In addition, health promoters were trained in educational modules. The educational module was developed based on WHO recommendations for physical activity in the general population [14, 38]. Additionally, adults with diabetes should be encouraged to reduce their total daily sedentary time [15, 16, 39].

Patients received a 30–50 min educational program aimed at following current international recommendations for the general population. The educational program includes (1) vigorous intensive work-related activities such as weightlifting, forestry (cutting, chopping, carrying wood), cutting crops, gardening (digging), grinding, laboring (shoveling sand), loading furniture (stoves, fridge) and cycle rickshaw driving and moderate-intensity work-related activities such as cleaning (vacuuming, mopping, polishing, scrubbing, sweeping, ironing), washing (beating and brushing carpets, wringing clothes (by hand), gardening, milking cows (by hand), planting and harvesting crops, digging dry soil (with spade), woodwork (chiseling, sawing softwood), mixing cement (with shovel), labouring (pushing loaded wheelbarrow and drawing water; (2) travel to and from places activities that include walking or bicycle (pedal cycle) and moving around throughout the day for at least 10 min continuously, brisk walking 30 min five days a week; (3) Recreation/leisure-time/-related activities includes vigorous-intensity sports, fitness or recreational activities such as running or football, tennis, aqua aerobics and fast swimming for at least 10 min continuously and moderate-intensive recreational activities such as cycling, dancing, horse-riding, yoga and team sports. All diabetics performed 150 min of moderate-intensity aerobic exercise, at least 30–50 min, 5–3 days per week respectively. In addition, patients should increase unstructured physical activity, such as housework activities.

Patients attended an educational session on the same day as their medication appointment. Three educational sessions were held each week. Educational sessions take the form of lectures, group discussions and sometimes individual consultations. The educational session approach aims to increase understanding of the proposed areas of physical activity and facilitate adoption of the proposed exercises. During follow-up, patients received written educational materials to help with practical management.

Additionally, the assigned educator performed the following key activities:

(1) Encourage safe and effective physical activity; (2) Assess physical and emotional barriers to regular physical activity programs; (3) work with individuals to develop appropriate action plans; (4) assist a person set goals for physical activity; (5) record the type, number of days, and duration of physical activity performed; and (6) encourage increased efforts to create safe spaces for physical activity and reduce barriers to physical activity in the community.

A control group did not receive any specific intervention during follow-up. Control patients received usual care according to national guidelines for non-communicable diseases. During follow-up, the study group was adhere to the patient’s care by the healthcare provider and study team as in the intervention group, except for the newly developed exercise package. All patients at the hospital received similar exercise package after the newly introduced package was scale up.

### Measurement of the outcome variables

Outcome parameters included changes in physical activity habits and practices and changes in glycemic control. Data were measured twice (baseline and end line survey after six months using interviewer-administered questionnaires). At each selected public hospital, two trained nurses collected all relevant data. During follow-up, the patient’s blood glucose was measured with a PRODIGY® blood glucose meter.

Physical activity data were collected using the WHO Physical Activity Questionnaire [38] and analyzed using the Global Physical Activity Questionnaire Analysis Guide [38]. This includes the types of activities they did, the time spent on each activity, and the number of days per week spent on each activity. A diabetic patient who reported moderately vigorous physical activity ≥ 5 days per week or vigorous physical activity ≥ 3 days per week met national guidelines for physical activity [38].

Fasting blood sugar (FBS) is the most commonly used measure of glycemic control. The patient was asked to fast at least 8 h prior to the appointment in order to provide a blood sample for laboratory testing. The mean of the FBS measurements for three consecutive months was used for the baseline FBS analysis. Mean values ​​of six consecutive months of FBS measurements were used for analysis during follow-up. Based on ADA guideline recommendations, glycemic status is classified as good if mean FBS is between 80 and 130 mg/dL and poor if above 130 mg/dL [40].

### Data management and statistical analysis

For statistical analysis, data were entered into Epi Data version 3.1 and exported to the Statistical Package for Social Sciences (SPSS) version 22. Continuous variables presented as the mean ± standard deviation and categorical variables presented as percentages. An intention-to-treat analysis was used to test the hypothesis. Between-group comparisons were made using independent-samples t-tests to assess the effect of interventions on physical activity practice. P-value ​​less than 0.05 was considered significant by all statistical tests.

## Results

### Soci-demographic and clinical characteristics of the patients

A total of 216 patients in both groups participated in the study. However, one patient was withdrawn from the intervention group after three months of follow-up. The majority of participants in the intervention and control groups were from urban, with 83 (76.9%) and 70 (64.8%) being married and having a formal education, respectively.

Both groups had approximately the same proportion of male and female participants. In the intervention and control groups, the mean time since DM diagnosis was 5.45 ± 4.16 years and 5.33 ± 5.11 years, respectively (Table 1).

**Table 1:** Demographic characteristics of patients in the intervention and control groups in the North Shao zone, 2021

Questions	Control (n = 108, %)	Intervention (n = 108, %)
**Age of the respondent**		
Young age group (15–47 years)	28 (25.9)	37(34.3)
Middle age group (48–63 years)	61 (56.5)	51 (47.2)
Elder age group (≥ 64)	19 (17.6)	20 (18.5)
**Sex of the respondent**		
Male	53 (49.1)	56 (51.9)
Female	55 (50.9)	52 (48.1)
**The residential area of respondents**		
Urban	83 (76.9)	70 (64.8)
Rural	25 (23.1)	38 (35.2)
**Marital status of the respondents**		
Married	83 (76.9)	71 (65.7)
other (Single, Divorced, Widowed)	25 (23.1)	37 (34.3)
**Educational status of the respondent**		
No formal education	35 (32.4)	35 (32.4)
The primary and high school completed	40 (37.0)	54 (52.8)
Attended higher education (certificate and above )	33 (30.6)	16 (14.8)
**Employment status of the respondents**		
Farmer	21 (19.4)	26 (24.1)
Housewife	36 (33.3)	26 (24.1)
Other (Gov’t employee, Private employee, and Merchant	51 (47.3)	56 (51.8)
Presence of comorbidity? (Yes)	38 (35.2)	23 (21.3)
Type of co-morbidity		
Hypertension	33 (86.8)	15 (65.2)
Asthma	4 (10.6)	5 (21.8)
Cardiovascular disease	1 (2.6)	3 (13.0)
Duration since diagnosis of DM (years), (Mean ± SD)	5.45 ± 4.16	5.33 ± 5.11
Duration since starting DM treatment (years), (Mean ± SD)	5.52 ± 5.21	4.31 ± 4.27

### Effect of physical activity promotion program on the adhere to recommended physical activities

The results showed that after the educational intervention, the mean scores of participating in moderate-intensity physical activity and taking time, walking for at least 10 min continuously and taking time, and participating in moderate intensity leisure activities and time spent time were increased, statistically significant differences between groups (p ≤ 0.05). However, there was no difference in time to participate in moderate-intensity recreational activities and perform high-intensity recreational activities between the two groups (p < 0.05) (Table 2).

**Table 2:** Comparison of the mean score of the recommended physical activities and time spent by patients between the two groups in North Shoa zone, 2021

Recommended physical activities	Type of group	After 6 months follow up	Independent t‑test		
			T	df	p-value
Exercising vigorous-intensity activities as part of your work	Intervention	2.52 ± 0.72	1.3	149	0.211
	Control	2.55 ± 0.51			
Time spend doing vigorous-intensity activities	Intervention	152.43 ± 55.91	0.5	148	0.613
	Control	136.32 ± 32.53			
Exercising moderate-intensity activities as part of your work	Intervention	3.70 ± 1.65	5.6	168.7	< 0.0001
	Control	2.53 ± 0.91			
Time spend doing moderate-intensity activities	Intervention	142.0 ± 51,04	2.4	183.4	0.017
	Control	129.10 ± 35.68			
Exercising walking or bicycle for at least 10 min continuously	Intervention	4.09 ± 1.71	8.5	152.3	< 0.0001
	Control	2.44 ± 0.82			
Time spent walking or bicycling for travel on a typical day	Intervention	152.94 ± 54.08	3.1	196	0.002
	Control	128.29 ± 47.7			
Exercising vigorous-intensity recreational (*leisure*) activities?	Intervention	2.63 ± 0.52	1.8	104	0.079
	Control	2.50 ± 0.71			
Time spend doing vigorous-intensity recreational activities	Intervention	123.63 ± 40.81	2.0	41.2	0.054
	Control	87.5 ± 38.62			
Exercising moderate-intensity recreational (*leisure*) activities	Intervention	2.66 ± 0.52	2.6	126	0.011
	Control	2.25 ± 0.60			
Time spend doing moderate-intensity recreational (*leisure*) activities	Intervention	114.36 ± 39.54	1.1	42.7	0.290
	Control	111.43 ± 41.40			
Overall physical activities score per week	Intervention	3.12 ± 0.65	7.6	201.2	< 0.0001
	Control	2.51 ± 0.52			
Overall time spent score for physical activities with a minute per week	Intervention	139.92 ± 24.33	2.3	207	0.023
	Control	131.68 ± 29.20			

### Adherence to the recommended physical activities

**Fig. 1:**
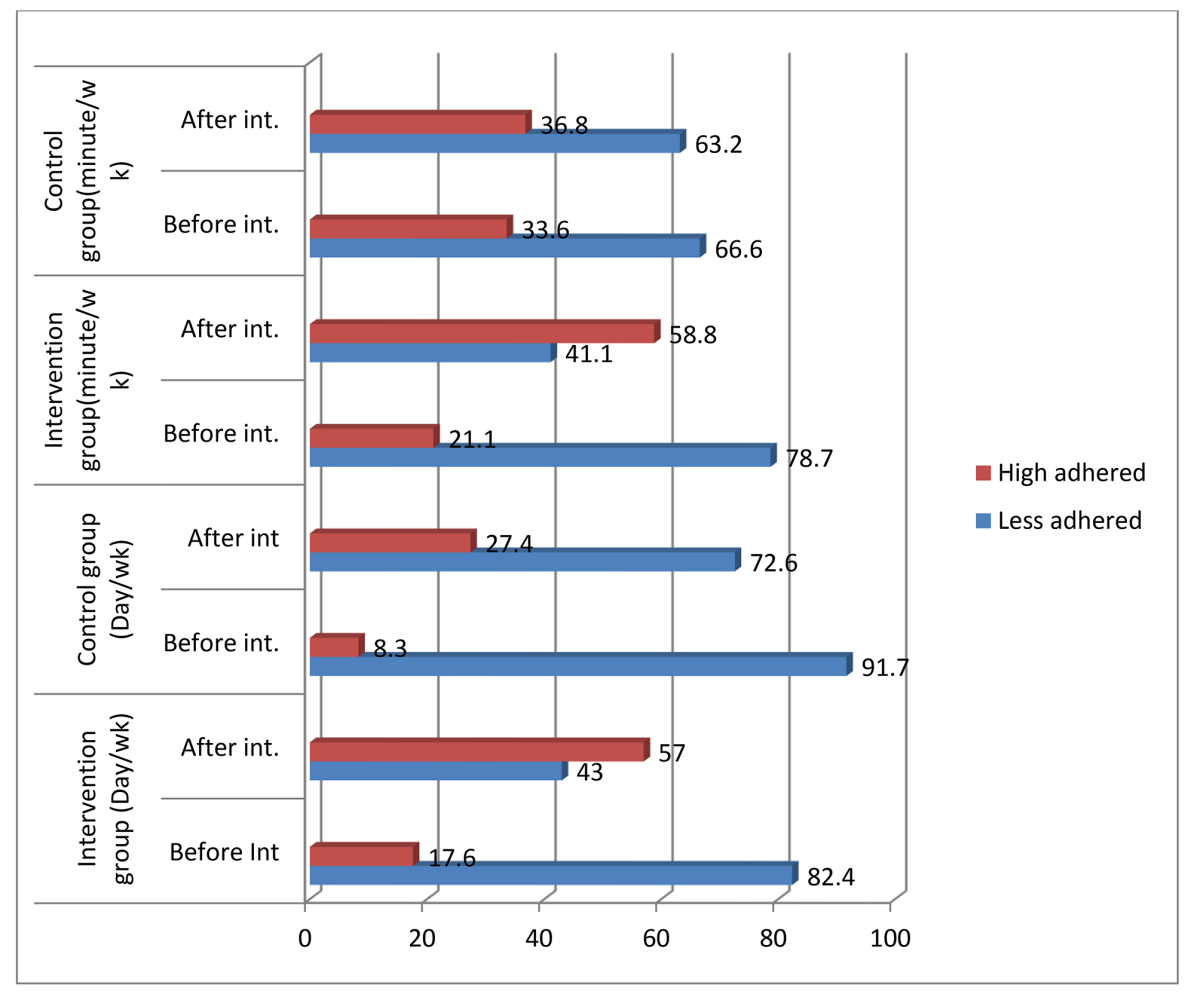
Proportion of the recommended physical exercises with day and time spent in minutes per week among groups in North Shoa zone, 2021

After six months of follow-up, the proportion of good exercise in terms of days and minutes per week was 61 (57%) and 63 (58.8%) in the intervention group and higher, respectively, than in the control group (Fig. 1). The intervention groups had a higher total number of days and minutes of physical activity per week (3.12 ± 0.65) than the control groups (2.51 ± 0.52). After the educational intervention, the independent t-test showed a statistically significant difference (p < 0.0001) between the groups (Table 2).

### The effect of physical activity promotion program on glycemic control

**Fig. 2:**
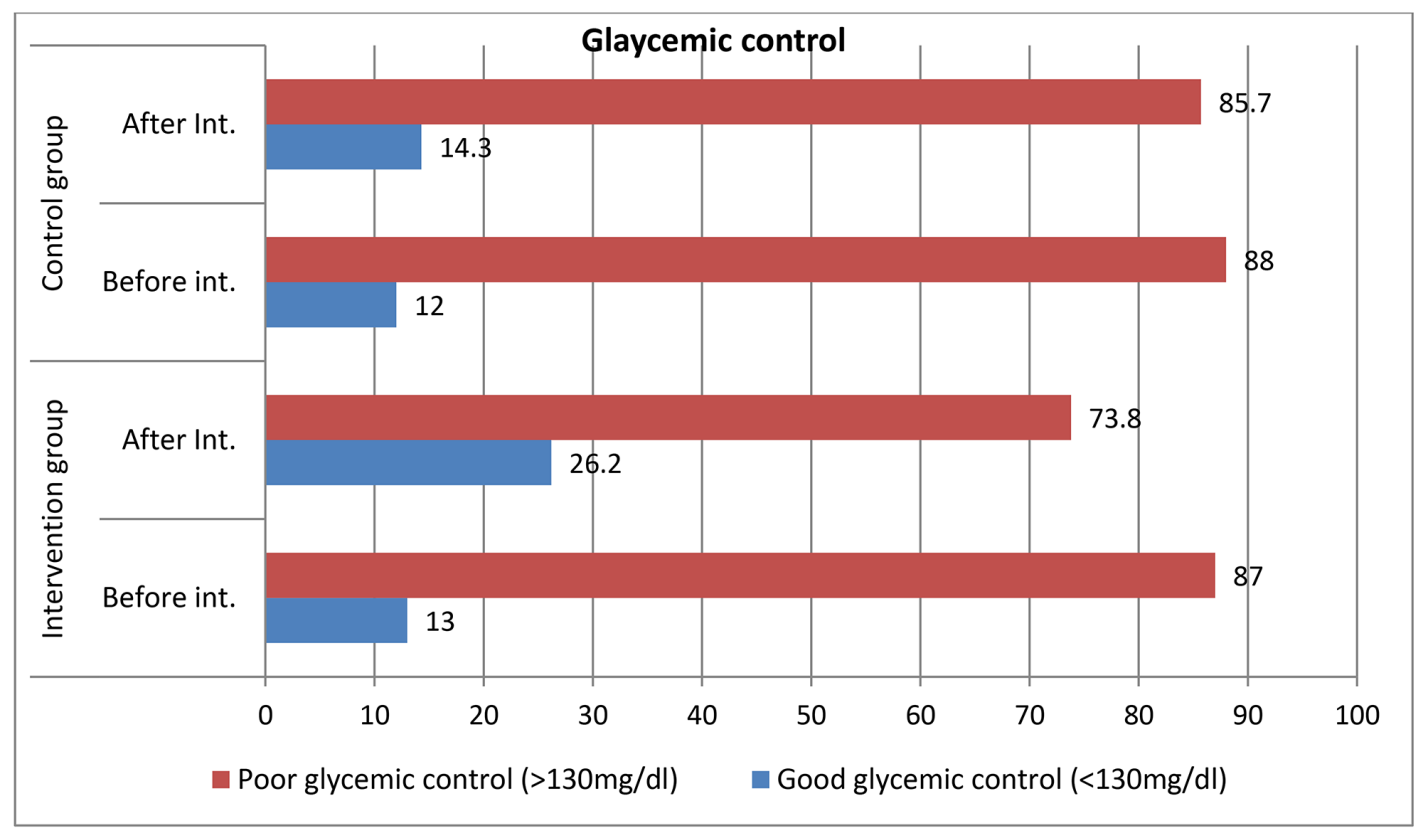
Proportion of glycemic control among groups in the North Shoa zone, 2021

The mean fasting blood glucose level after educational intervention was 168.76 ± 49.57 mg/dl in the intervention group and 182.40 ± 42.91 mg/dl in the control group. Mean fasting blood glucose levels showed a statistically significant difference between groups by independent t-test (p = 0.033) (Table 3). After 6 months of intervention, the proportion of good glycemic control was 28 (26.2%) in the intervention group and 15 (14.3%) in the control group (Fig. 2).

**Table 3:** Comparison of the mean score of FBS among the groups before and after the intervention in the North Shoa zone, 2021

Variable	Type of group	Mean ± SD		Paired t-test
		Before the intervention	After 6 months follow up	p-value
FBS level	Intervention group	187.72 ± 59.67	168.76 ± 49.57	0.008
	Control group	184.44 ± 51.44	182.40 ± 42.91	0.727
	Independent t‑test ( p-value)	-	0.033	-

## Discussion

The aim of the current study was to examine the effects of a physical activity promotion program on physical activity adherence and glycemic control. In this study, promotion of a physical activity program increased the average number of days and duration of physical activity per week by 3.12 ± 0.65 and 139.92 ± 24.33 min, respectively, with statistically significant differences between groups after the intervention (p < 0.0001).

This finding was consistent with previous studies showing a statistically significant increase in adherence to physical activity levels with different behavioral intervention programs (p < 0.05) [23–26]. According to published studies, increasing physical activity and reducing sedentary behavior are successful public health strategies for the management and prevention of type 2 diabetes [23, 29, 41]. However, according to a systematic review conducted in Ethiopia, physical activity/exercise is a low-priority lifestyle choice in society[13].

Educational interventions aimed at promoting behavior change towards physical activity are (1) targeted to physical activity, (2) designed to use established behavior change techniques, and (3) mobilized social support are more likely to be effective for maintaining physical activity. Therefore, health care providers and health educators should carefully consider including the above elements of intervention programs in interventions to maximize the effectiveness of interventions that promote changes in physical activity [42, 43]. Focusing on physical activity barriers can also help improve physical activity habits [27, 28, 44–46]. Furthermore, physical activity habits can be associated with access to appropriate individualized programs, improved availability and accessibility of community facilities, creation of clear relationships between patients and health care providers, and improved patient attitudes towards learning [27, 28, 44–46].

This current study increased mean scores for participation in moderate-intensity and duration activity, at least 10 min of continuous walking and time spent, and engaging in moderate-intensity and duration leisure activities increased, and showing a statistically significant difference between groups (p < 0.05). These findings are supported by previous studies in which educational interventions significantly increased adherence to recommended exercise [26, 47]. A diabetic should do 150 min of moderate to vigorous aerobic exercise, 3–5 times a week for 30 min, with no more than two days off. People with type 2 diabetes should do strength training at least 2–3 times per week, reduce sedentary behavior, and increase daily unstructured physical activity [29, 30].

This study examined the effect of a physical activity program on glycemic control in people with type 2 diabetes. Results of this study showed that mean fasting blood glucose levels were significantly lower after the educational intervention (p = 0.033). This finding is supported by previous studies [16, 23, 24, 39, 48] that found that educational interventions significantly reduced fasting blood glucose levels.

This study has the following limitations; results are based on patient responses to assessments of activity/exercise changes that contribute to information bias. Social desirability bias may have influence on the results. Also, having multiple teaching methods means that it cannot know which method makes a difference in the study group.

### The implication of the study

Implementing a physical activity program is a preferred strategy in diabetes management. This has been demonstrated in previous studies [23–26] and in this study. To support patient self-management behaviors, recommended diabetes interventions should be integrated into community-based health care and take into account the patient’s cultural context. Thus, primary care platforms such as health posts and health centers can play an important role in integrating health promotion programs to improve self-management behaviour. Comprehensive and timely patient-centered intervention strategies are needed at the community and household levels to improve self-management behavior. This can be applied by integrating diabetes management interventions into community health services (health extension packages).

In summary, policymakers and providers are focusing on the following key program areas: 1) Physical activity promotion programs should be integrated into existing systems as a common therapeutic service /treatment/.2) Design different strategies to create health promotion programs to reach large communities in need using a wide range of learning strategies. 3) Assign a health promoter to provide and design a health education program on physical activity recommended as routine care. 4) Focus on a detailed analysis and understanding of the barriers faced by the patient and integrate them into their daily activities. Furthermore, healthcare providers should understand their patients’ basic needs and encourage them to form community clubs, such as walking clubs.

## Conclusions

In this recent study, a physical activity promotion program increased the mean scores of exercising moderate-intensity activities and spending time (p < 0.05), continuous walking for at least 10 min and spending time (p < 0.05), and moderate-intensity recreational activities and spending time (p < 0.05). Overall, the physical activity program increased levels of adherence to recommended physical activity and time spent and showed a statistically significant (p ≤ 0.05). The educational intervention significantly reduced mean fasting blood glucose levels (p ≤ 0.05).

## Data Availability

All data generated in this study are included in the manuscript. The dataset is available upon reasonable request from the corresponding author.

## Abbreviations

ADA: American Diabetes Association
DM: Diabetes Mellitus
FBS: Fasting Blood Glucose
SPSS: Statistical Package for Social Sciences
SD: Standard Deviation
T2DM: Type II Diabetes Mellitus
